# Tracking of Adipose-Derived Mesenchymal Stromal/Stem Cells in a Model of Cisplatin-Induced Acute Kidney Injury: Comparison of Bioluminescence Imaging versus qRT-PCR

**DOI:** 10.3390/ijms19092564

**Published:** 2018-08-29

**Authors:** Ralf Schubert, Julia Sann, Jochen T. Frueh, Evelyn Ullrich, Helmut Geiger, Patrick C. Baer

**Affiliations:** 1 Division of Allergology, Pneumology and Cystic Fibrosis, Department for Children and Adolescents Medicine, Hospital of the Goethe-University, 60596 Frankfurt, Germany; ralf.schubert@kgu.de; 2 Division of Nephrology, Department of Internal Medicine III, Goethe-University, 60596 Frankfurt, Germany; julia.sann@t-online.de (J.S.); h.geiger@em.uni-frankfurt.de (H.G.); 3 Division for Stem Cell Transplantation and Immunology, Department for Children and Adolescents Medicine, Hospital of the Goethe University Frankfurt, 60590 Frankfurt, Germany; Jochen.Frueh@kgu.de (J.T.F.); Evelyn.Ullrich@kgu.de (E.U.); 4 LOEWE Center for Cell and Gene Therapy, Goethe University, 60590 Frankfurt, Germany

**Keywords:** tracking, renal failure, acute kidney injury, mesenchymal stromal/stem cells, bio imaging, bioluminescence, qRT-PCR, PCR

## Abstract

Determining the cell fate and the distribution of mesenchymal stromal/stem cells (MSCs) after transplantation are essential parts of characterizing the mechanisms of action and biosafety profile of stem cell therapy. Many recent studies have shown that MSCs migrate into injured tissues, but are only detectable at extremely low frequencies. We investigated the cell fate of MSCs after transplantation in an acute kidney injury (AKI) mouse model using in vivo bioluminescence imaging (BLI) and subsequent verification of cell migration using quantitative real-time polymerase chain reaction (qRT-PCR). The AKI was induced by a single injection of cisplatin (8 or 12 mg/kg). One day later, adipose-derived mesenchymal stromal/stem cells isolated from luciferase transgenic mice (Luc^+^-mASCs, 5 × 10^5^) were intravenously transplanted. Migration kinetics of the cells was monitored using BLI on day 1, 3, and 6, and finally via quantitative real-time PCR at the endpoint on day 6. Using BLI, infused Luc^+^-mASCs could only be detected in the lungs, but not in the kidneys. In contrast, PCR endpoint analysis revealed that Luc-specific mRNA could be detected in injured renal tissue; compared to the control group, the induction was 2.2-fold higher for the 8 mg/kg cisplatin group (*p* < 0.05), respectively 6.1-fold for the 12 mg/kg cisplatin group (*p* < 0.001). In conclusion, our study demonstrated that Luc-based real-time PCR rather than BLI is likely to be a better tool for cell tracking after transplantation in models such as cisplatin-induced AKI.

## 1. Introduction

Determining the cell fate and the distribution of mesenchymal stromal/stem cells (MSCs) after transplantation are essential parts of characterizing the mechanisms of action and biosafety profile of stem cell therapy [1]. The therapeutic use of transplanted MSCs by intravenous (IV) administration was investigated by in vivo studies and injury models. These in vivo studies used various techniques to label and track culture-expanded and transplanted MSCs in different tissues over time, e.g., cell labeling by fluorescence or radioactivity, transfection with reporter genes (luciferase or green fluorescent protein), or the use of donor cell-specific markers (human Alu sequence) [1,2,3,4,5,6,7]. These studies have shown that transplanted MSCs distribute to a variety of injured tissues, but, in many cases, are still poorly detectable in the potential target tissue at later time points. After administration of the MSCs, a very large proportion of the cells initially accumulated in the lungs, followed by the liver and spleen [3]. Though many cells initially localize in the lungs as the major capillary bed encountered after systemic administration, the overall donor cell retention has been shown to be minimal [8], probably also due to the detection limit of the method used.

Mesenchymal stromal/stem cells are described as immature cells within the bone marrow, within nearly all adult vascularized tissues (e.g., adipose tissue, synovium, dermis) and in solid organs (e.g., liver, spleen, lung) [9,10]. Mesenchymal stromal/stem cells are a rare and quiescent population (or populations) in the perivascular niche (or are derived from perivascular cells or pericytes [11,12]) within fully specialized tissues. The cells are described as multipotent cells, can differentiate into specialized cells, including bone, cartilage, muscle, and epithelial cells, and have been successfully used in many regenerative approaches. Treating kidney injury, for example, in small animal models suggested that MSCs are a potential therapy option to support renal regeneration [13,14,15]. Whereas the kidney has a remarkable capacity for regeneration after acute kidney injury (AKI) and may recover completely, the options of clinical intervention are currently restricted to fluid management and dialysis. Thus, the development of new strategies to improve renal regeneration after AKI is urgently needed. Stem cell-based therapy seems to be a promising option in the treatment of AKI that requires cells with a maximum capacity to support renal regeneration. Several studies have shown a relatively low number of MSCs engrafting in the kidney and have already described significant renoprotective effects of MSCs [16]. In particular, tracking of transplanted MSCs and their distinct localization to the kidneys has been shown by bioluminescence imaging (BLI) in mice with ischemia- and reperfusion-induced AKI [5]; however, not yet in a toxic AKI model. In the present study, we investigated the cell fate of MSCs after transplantation in an AKI model using in vivo BLI and subsequent verification of luciferase mRNA in the kidney using quantitative real-time reverse transcriptase-PCR (qRT-PCR).

## 2. Results

### 2.1. Characterization of mASC

**Figure 1:**
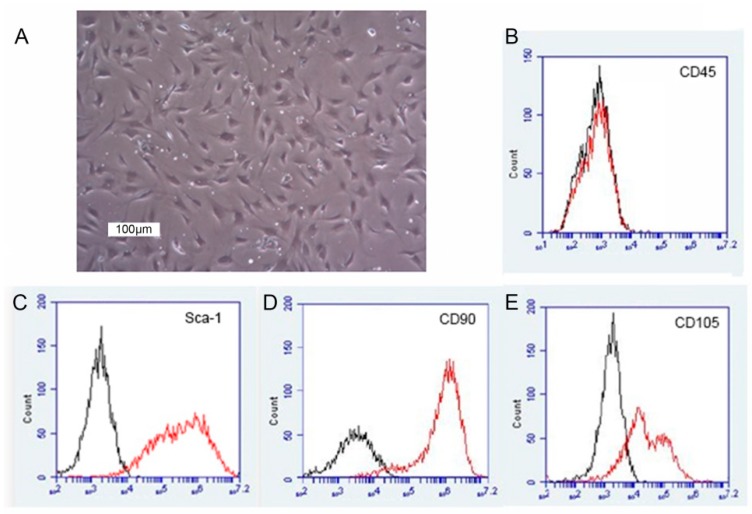
Characterization of luciferase-transgene mesenchymal stem cells from murine inguinal adipose tissue in passage 3 (Luc^+^-mASCs). (**A**) Phase contrast microscopy and surface expression of characteristic antigens of Luc^+^-mASCs in primary cell culture. (**B**–**E**) Representative flow cytometric histograms of the markers CD45-FITC (**B**), Sca-1-APC (**C**), CD90-PE (**D**), and CD105-APC (**E**). Unstained cells served as negative control (black lines).

Cells were characterized by morphological analysis and flow cytometric analysis of characteristic markers for murine mesenchymal stromal/stem cells. Cultured Luc^+^-mASCs displayed a characteristic spindle-shaped fibroblastoid morphology in culture (Figure 1A). Cultured Luc^+^-mASCs were shown to express Sca-1, CD90, and CD105, but did not express CD45 (Figure 1B–E). The morphology and expression pattern of these ASCs were comparable with studies from our group [17,18] and from other groups [19].

### 2.2. In Vitro Bioluminescence and PCR Analysis

**Figure 2:**
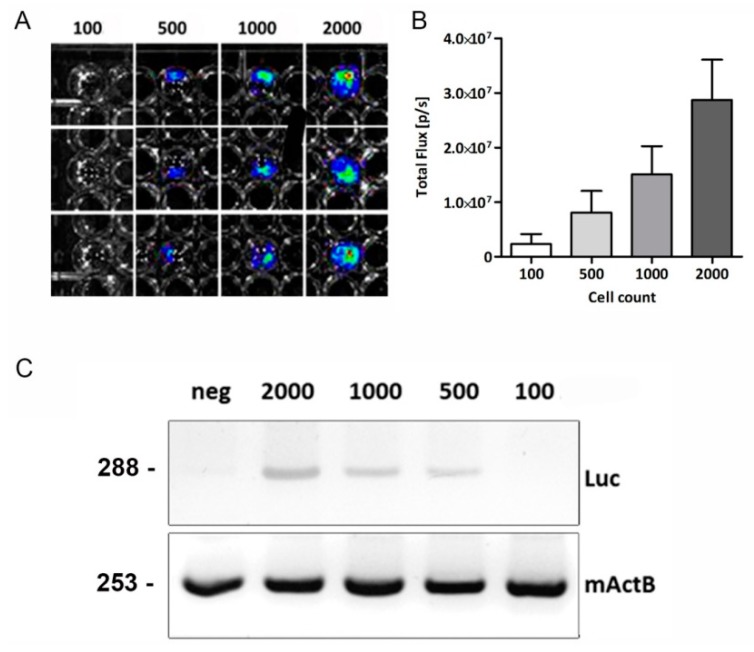
In vitro evaluation of Luc^+^-mASCs. (**A**) Bioluminescence signal of Luc^+^-mASCs after addition of d-Luciferin in culture (in triplicate: 100, 500, 1000, and 2000 cells). (**B**) Quantification of the bioluminescence signal (Photons/s) in relation to the total number of cells (*n* = 6). (**C**) Sensitivity of conventional PCR, amplified with specific primer for luciferase and murine β-actin (mActB) as a housekeeping gene. Determination of the detection limit of luciferase RNA using PCR and several dilutions of RNA transcripts (Neg = RNA from 100,000 Luc^-^-mASCs, and dilutions: 2000 Luc^+^-mASCs + 98,000 Luc^-^-mASCs; 1000 Luc^+^-mASCs + 99,000 Luc^-^-mASCs).

The Luc-transgene mice express the Luc reporter gene constitutively in nearly all tissues and organs, except red blood cells. The Luc^+^-mASCs were seeded in 96-well plates in an amount ranging from 1 × 10^2^–2 × 10^3^ cells to test the sensitivity of BLI in vitro. Representative bioluminescence images of Luc^+^-mASCs are shown in Figure 2A. The detection limit was around 500 Luc^+^-mASCs (Figure 2A), and there was no detectable signal of 100 Luc^+^-mASCs (Figure 2A). In vitro analysis showed a linear relationship between cell numbers versus BLI signals (as described earlier by Tögel et al. [5]). These data suggest that BLI of Luc^+^-mASCs can be used to follow and quantify transplanted stem cells accurately in small living animals.

Furthermore, we established PCR analysis of luciferase RNA to detect Luc^+^-mASCs in vivo. The specificity of the PCR luciferase analysis, documented by a gel electrophoreses image (Figure 2C), resulted in a single product with the desired length (Luc 288 bp, mActB 253 bp) (Figure 2C). The detection limit was around 500 Luc^+^-mASCs diluted in 1 × 10^5^ WT-mASCs (Figure 2C). Therefore, we could detect a single Luc^+^ cell in 200 WT-mASCs. Similar to the BLI assay described above, we could not detect a signal of 100 Luc^+^-mASCs diluted in 1 × 10^5^ WT-mASCs (Figure 2C). In addition, a LightCycler melting curve analysis was performed, which resulted in single product-specific melting temperatures (Figure S2). No primer-dimers were generated during the 40 qRT-PCR amplification cycles applied (data not shown). The qRT-PCR efficiencies were calculated as described earlier [20,21]. The genes investigated showed high qRT-PCR efficiency rates (Luc, E = 1.9399; mActB, E = 1.9164) in the range investigated from 0.01 to 1.0 ng cDNA input (*n* = 3) with high linearity (Pearson correlation coefficient *r* > 0.95).

### 2.3. Cisplatin-Induced AKI

**Figure 3:**
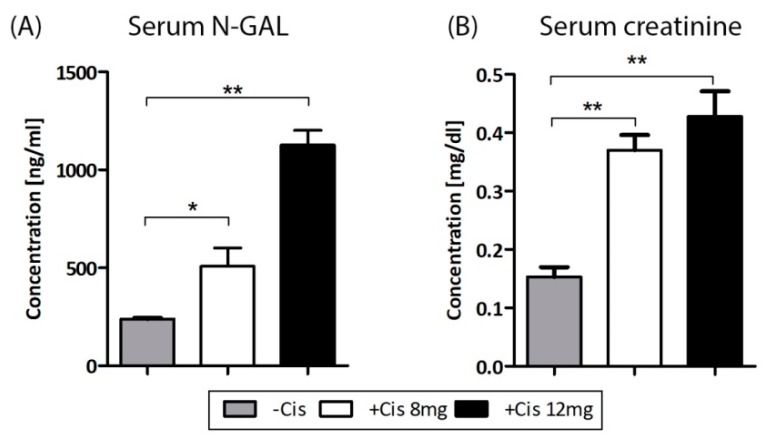
Effect of cisplatin injection on serum N-GAL (**A**) and creatinine (**B**) levels. Mice were injected with 8 mg/kg and 12 mg/kg cisplatin i*.p*. and serum levels of N-GAL and creatinine were measured on day 6. Data are expressed as ng/mL (N-GAL) and mg/mL (creatinine) (mean ± SEM); * *p* < 0.05 and ** *p* < 0.01 vs. control; *n* = 5 per group.

The levels of serum murine N-GAL (lipocalin-2) and serum creatinine, markers indicating altered renal function, were assessed after six days of cisplatin injection. In vivo cisplatin injection induced higher serum N-GAL and creatinine levels significantly compared to the buffer-injected control (Figure 3A,B). The most significant effect was observed in the group of 12 mg/kg cisplatin injection, whereas both cisplatin groups had significantly increased serum N-GAL and creatinine levels.

### 2.4. In Vivo Biolumunescence Imaging

**Figure 4:**
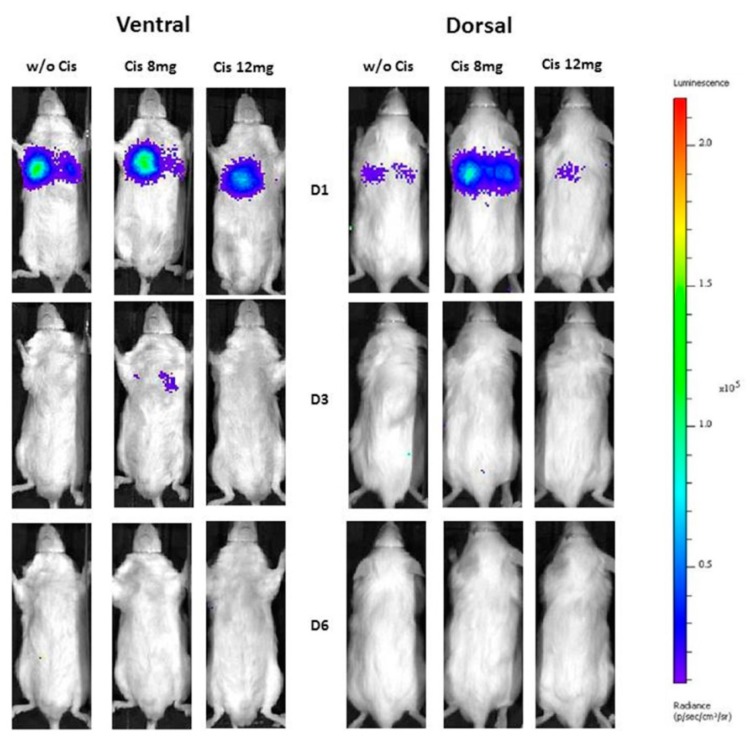
Bioluminescence imaging. Bioluminescence imaging measurements were performed on day 1, 3, and 6 to assess this biodistribution of transplanted Luc^+^-mASCs. Mice were imaged dorsally and ventrally. Representative animals of each group are shown (controls (w/o Cis) *n* = 8, Cis 8 mg, *n* = 10; Cis 12 mg, *n* = 6).

The current study using BLI was designed to track mASCs after IV injection in mice with cisplatin-induced AKI, and to investigate their distribution and survival kinetics over time. The BLI measurements were performed on day 1, 3, and 6 to assess this biodistribution of transplanted Luc^+^-mASCs (Figure 4). The mice were imaged dorsally and ventrally. The region of interest (ROI) was created over the thorax and average radiance/total flux was measured. In this setting, infused Luc^+^-mASCs could only be detected in the lungs of the animals, but not in the kidneys (Figure 4). Furthermore, we did not detect long-term engraftment of the transplanted cells. The BLI demonstrates that intravenously delivered mASCs accumulate preferentially to the lungs. Elevated ventral and dorsal signals in the lungs could be observed for all groups only on day 1, followed by total decrease in signal intensity on day 3 to 6 (Figure 4). We could not detect any BLI signals using ex vivo images of the removed lungs and kidneys on day 6 (Figure S1).

### 2.5. Endpoint qRT-PCR

**Figure 5:**
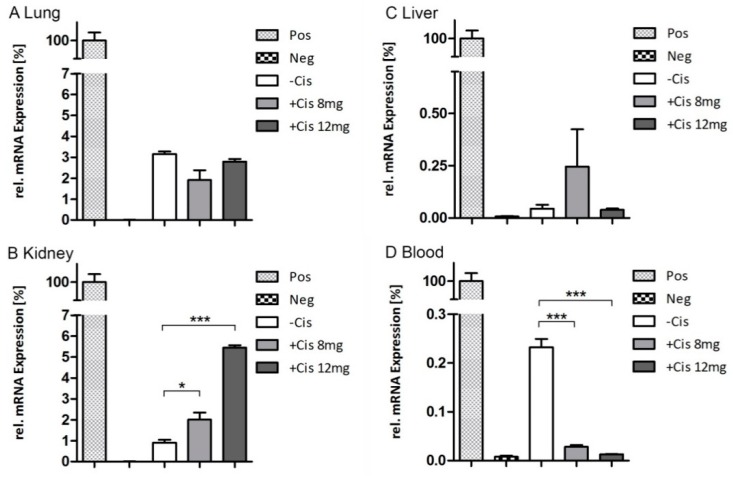
Endpoint qRT-PCR. PCR for luciferase expression was used to detect cells in (**A**) lung, (**B**) kidney, (**C**) liver tissue, and (**D**) blood in control mice and in AKI-induced mice at day 6 after cell injection. RNA from Luc^+^- and Luc^-^-mASCs was used as positive (Pos) and negative (Neg) control. * *p* < 0.05 and *** *p* < 0.001 vs. control; *n* = 5 per group.

We performed endpoint qRT-PCR analysis to determine whether there were Luc^+^-mASCs (Luc-specific mRNA) remaining in the organs that were below the BLI detection limit or to confirm the imaging results of day six. The PCR for luciferase expression was used to detect these remaining cells in RNA extracts from kidney, lung, liver tissue and blood on day six after cell injection in control mice and in mice with induced AKI (Figure 5). The relative efficiency-corrected mRNA expression of the target gene was calculated based on efficiencies € and the CT (Threshold cycle) deviation of an unknown sample versus a control, and expressed in comparison to a reference gene. In contrast to BLI, we could detect measurable amounts of relative mRNA expression at the endpoint of the study (day six). No significant differences between the treatment and the control group could be detected in the lungs (w/o Cis 3.15 ± 0.2%, Cis 8 mg 1.9 ± 0.82%, Cis 12 mg 2.79 ± 0.22%) (Figure 5A). Nevertheless, the luciferase expression values showed significantly higher levels in the kidneys of cisplatin-treated animals versus the control group (w/o Cis 0.9 ± 0.24%, Cis 8 mg 2.0 ± 0.58%, Cis 12 mg 5.5 ± 0.21%) (Figure 5B). Compared to the control group, the induction was 2.2-fold higher for the 8 mg/kg Cis group (*p* < 0.05), and respectively 6.1-fold for the 12 mg/kg Cis group (*p* < 0.001). In contrast, we detected lower blood luciferase mRNA levels in the animals with cisplatin-induced AKI than in control animals (*p* < 0.001).

## 3. Discussion

Laboratories worldwide have used a wide range of techniques in attempts to determine the distribution of infused cells in several injury models. Various methods such as in vivo BLI, magnetic resonance imaging, immunohistochemistry, PCR, and flow cytometry have been used to track the distribution of transplanted MSCs [2,5,6,7,22,23,24], whereas only the first two are suitable for real in vivo tracking. The main common results of recent studies to track IV-infused MSCs were that (1) MSCs distribute to a variety of tissues after injection; (2) MSCs are detectable at low frequencies in injured target tissues after transplantation; and (3) signals from the injected MSCs were found initially at the highest frequencies in the lungs [3].

In recent years, in vivo BLI and tracking based on luciferase activity or fluorescence molecules has gradually become the most favorable strategy to track transplanted MSCs in different animal models. Bioluminescence imaging has been successfully used to obtain semiquantitative measurements because of a strong correlation between the number of transplanted cells and the BLI signal detected both in vitro and in vivo [6]. Nevertheless, the sensitivity of BLI to detect these cells in vivo remains a major concern, because determining cell migration to injured renal tissue remains a challenge due to very low numbers of migrated cells. Furthermore, several other limitations of BLI measurements remain. These limitations not only depend on the expression level of the luciferase in infused cells and the rate of luciferin diffusion into the tissue, but also on the depth and optical density of the imaged tissue. It has been described that there is a 10-fold decrease in bioluminescence signal intensity for every centimeter of tissue depth [25]. In addition, cisplatin-induced AKI triggers renal vascular constriction which may hinder access of injected luciferin into the kidney, and therefore reduced the BLI signal in our setting. Nevertheless, Tögel and co-workers [5] demonstrated that BLI is a suitable tool for in vivo tracking of 1 × 10^5^ cells in an injury model of renal ischemia and reperfusion. Unfortunately, we could not detect infused Luc^+^-mASCs in injured kidneys by BLI in our model, although we infused 5 × 10^5^ Luc^+^-mASCs. We imaged the mice from the dorsal and the ventral side to test the sensitivity of BLI in our setting, but without a significantly different result. In our model settings, the BLI measurements only demonstrated that intravenously delivered cells accumulate in the lungs in all mice. Nevertheless, when using endpoint ex vivo qRT-PCR on day six, we could detect Luc-mRNA (from migrated mASCs, extracellular vesicles from mASCs or free Luc-specific RNA products, as discussed later) in the renal tissue of mice with cisplatin damage. In return, we could also show that the control animals had significantly higher levels of mASCs in the blood (compared to cisplatin-treated mice) by using qRT-PCR analyses, whereas the amount of remaining mASCs in the lungs was nearly similar in the controls and mice with induced AKI.

Other recent studies indicated that MSCs home to injured renal tissue, but only at very low levels after transplantation. It has been demonstrated that IV transplanted MSCs were found in the kidneys in a mouse model of acute renal failure caused by intramuscular injection of glycerol [13]. Others also performed IV injections in a mouse model of cisplatin-induced AKI and detected previously fluorescence-labeled and transplanted MSCs in the kidney [26]. These results suggest that peripheral IV transplantation is an effective method of cell therapy in renal diseases. Nevertheless, the site of cell application may bias the biodistribution of infused cells. The therapeutic effect of MSC transplantation in kidney disease has been achieved after IV intraarterial, renal subcapsular or i.*p*. delivery. Moustafa and co-workers [27] described that the route of the injected MSCs does not have a major influence on the outcome in an AKI model. It is well described that accumulation of MSCs in the lungs after IV delivery is a key determining factor for their biodistribution [28], and therefore their therapeutic effect [1,5,28]. Lee and co-workers [4] described that up to 80% of IV transplanted cells accumulate in the lungs. In our setting, infused Luc^+^-mASCs could also only be detected in the lungs of controls and AKI mice. Furthermore, the signal in the lungs decreased gradually from day one to three. Others also described that the retention of infused MSCs in the lung decreased rapidly within 24 h, and completely disappeared within four days [4]. Although we could not detect Luc^+^ cells in injured renal tissue using BLI, we detected Luc-mRNA by qRT-PCR analysis on day six. Our study clearly demonstrates that higher levels of Luc-mRNA were detectable in the injured kidneys when the grade of cisplatin-induced renal injury rose (as shown by N-GAL and creatinine measurements). In this case, it should be mentioned that this signal could also be generated from free Luc-mRNA rather than migrated mASCs. The RNA products derived from eliminated mASCs trapped in the lungs might circulate in blood, and be at least detectable in the kidneys after renal elimination. Therefore, Luc-specific RNA detected by real-time PCR might be derived from the dead cells eliminated from lungs. With the methods used here, we are not able to exclude that possibility. Nevertheless, ribonucleases are common in the serum and catalyze degradation of RNAs resulting in very short lifespans for any free RNA that is not in the protected cytosolic environment. The levels of serum RNase activities have been noticed to increase during kidney injury and correlate well with creatinine clearance [29,30]. Furthermore, we only detected a higher Luc-signal in cisplatin-injured kidneys, and not in kidneys of control animals. In the control animals, we detected a higher signal in the blood, which points to an injury-specific event and contradicts the possibility of free Luc-specific RNA. On the other hand, the Luc-specific PCR proof may also be generated by extracellular vesicles or exosomes released from transplanted Luc^+^-mASCs. Extracellular vesicles and exosomes have been shown as a new mechanism of cell-to-cell communication that allows the transfer of functional proteins, mRNAs, and micro-RNAs upon cell activation, and also play a pivotal role during renal regeneration after MSC transplantation [31].

The therapeutic action of MSCs during kidney injury has also been reported by several other groups. Ezquer and co-workers [32] investigated whether renoprotection of injected MSCs is related to the recruitment of donor cells into the kidneys. They evaluated the biodistribution of transplanted MSCs and their effect to prevent renal failure in diabetic mice [32]. In this study, donor cells were shown in the kidneys of diabetic mice three months after transplantation, although at very low levels, using a flow cytometric technique to proof migrated green fluorescence protein-positive MSCs. Morigi and co-workers [33,34] have shown that treatment with MSCs in cisplatin-induced AKI maintained renal function and protected against tubular injury. In this regards, they used in situ hybridization to detect the Y chromosome of transplanted MSCs isolated from male donor mice in the injured kidneys of female recipients [34]. In addition, several other studies demonstrated that MSCs localize within injured renal tissue after transplantation in mice with AKI [reviewed in [35]). The results obtained from different models of AKI and chronic kidney disease document that MSCs have a huge therapeutic potential in renal regeneration, preserving renal function. The mechanisms underlying this process triggered by MSCs transplantation seem to refer mainly to their paracrine activity. The MSCs are described as migrating to the site of injury and secreting a pool of growth factors and cytokines with anti-inflammatory, immunomodulatory, anti-apoptotic, and survival-promoting effects [14]. Nevertheless, we did not prove amelioration of renal regeneration or renoprotection after cell transplantation in our recent study. The current study was only designed to track the MSCs after IV injection in mice with induced AKI and to test the sensitivity of in vivo BLI compared to ex vivo Luc-based qRT-PCR in this setting. In conclusion, our study demonstrates that Luc-based qRT-PCR rather than BLI of Luc^+^-mASCs is likely to be a better tool for cell tracking after transplantation in models such as cisplatin-induced AKI.

## 4. Materials and Methods

### 4.1. Animals

We used 12-week-old female Albino C57BL/6 mice (Janvier, Saint-Berthevin Cedex, France) for the BLI. We used luciferase transgene B6-L2G85 mice for the cell isolation [36,37]. Mice were housed in plastic cages on a 12-h light/12-h dark cycle with access to food and water ad libitum until harvest. All animal procedures were approved by the Animal Care and Use Committee of the state of Hessen (RP Darmstadt, permit number F61/19, 16 July 2012). Animal care and procedures were performed in accordance with the “Guide for the Care and Use of Laboratory Animals” (NIH, volume 25, no. 28, revised 1996), EU Directive 86/609, and the German Animals Protection Act.

### 4.2. Cell Isolation, Culture, and Characterization

Luciferase transgene adipose-derived stromal/stem cells (Luc^+^-mASCs) were harvested from inguinal adipose tissue, as previously described [17,18]. The mASCs from non-transgene, wild type mice were isolated as control cells (WT-mASCs) for selected experiments. Dulbecco’s modified Eagle’s medium (DMEM, Sigma, Taufkirchen, Germany) with a physiologic glucose concentration (100 mg/dL) was supplemented with 10% fetal calf serum (Biochrom, Berlin, Germany) and used as a standard culture medium. Cells between passage 2 and 5 were used throughout the experiments.

Cell morphology was examined by phase contrast microscopy. Flow cytometric analysis was carried out to show their characteristic marker expression. Cells (passage 3) were detached by Trypsin/EDTA solution from culture plastic and stained with directly labeled antibodies (CD90-PE: Southern Biotech, Birmingham, AL, USA, No. 1750-09, CD105-APC: Biozol, Eching, Germany No. 120413, Sca-1-APC: www.eBioscience.com, No. 17-5981-81, and CD45-FITC: Immunotools, Friesoythe, Germany, No. 22150453). The labeled cells were analyzed using a BD Accuri C6 flow cytometer (BD Biosciences, Heidelberg, Germany). All experiments included negative controls with isotype controls.

### 4.3. In Vivo Model of Cisplatin-Induced Acute Kidney Injury

We used the chemotherapeutic agent cisplatin to induce an AKI in vivo. Therefore, a single intraperitoneal (i.*p*.) injection of cisplatin (8 or 12 mg/kg body weight, Teva Pharmaceuticals Ltd., Petach Tikva, Israel) was administered on day 1 (8 mg/kg, *n* = 10; 12 mg/kg, *n* = 6) (Figure S2). The control mice received saline solution i.*p*. (*n* = 8). On day 0 (= day 1 after induction of AKI), 5 × 10^5^ Luc^+^-mASCs in DMEM containing Heparin (10 U/100 µL) were transplanted. Viability of the cells was checked by trypan blue exclusion immediately before transplantation. A single injection of 100 µL was conducted via the tail vein into each mouse. Cells were tracked via BLI on day 1, 3, and 6, and via qRT-PCR at the endpoint on day 6 (Figure S2). The mice were weighed every two days. Blood samples were collected from the vena facialis using the microvette capillary blood collection system (Sarstedt, Nümbrecht, Germany) on day 6. The mice were sacrificed at the end of the experiment by cervical dislocation under anesthesia with ketamine-xylazine, and the kidneys were collected. 

We used a commercially available creatinine assay kit (Abcam, Cambridge, UK) for the colorimetric evaluation of serum creatinine. We also checked serum neutrophil gelatinase-associated lipocalin (N-GAL/lipocalin-2) values, a biomarker of kidney injury, of each mouse in the experiments on day 6 by an immunoassay for murine N-GAL/lipocalin-2 (BioPorto Diagnostics, Hellerup, Denmark), according to the manufacturer’s protocol. Serum samples were diluted 1:2000 (N-GAL) or used undiluted (creatinine). Detection was performed using a Dynatech MR 5000 microplate reader (Dynex Technologies GmbH, Denkendorf, Germany).

### 4.4. Polymerase Chain Reaction

Total RNA was isolated from kidney, lung, and liver tissues and blood immediately after collection, or from cultured cells directly within the culture dish. The RNA extraction was performed using a modified protocol of Chomczynski and Sacchi [38]. After RNA extraction and synthesis of cDNA by using reverse transcriptase, qRT-PCR was carried out using a mixture of SYBR Green I fluorescence dye with the following conditions: 15 min at 95 °C for enzyme activation, then 40 cycles of 15 s at 95 °C for denaturation, 60 s at 63 °C for annealing, and 30 s at 72 °C for elongation. Primer pairs were synthesized by Invitrogen (Karlsruhe, Germany). The following primers were used: murine β-actin (NM_007393, forward CCACCATGTACCCAGGCATT, reverse AGGGTGTAAAACGCAGCTCA, product size 253 bp), firefly luciferase (GenBank No. AB644228.1, forward TGAAGAGATACGCCCTGGTT, reverse CTACGGTAGGCTGCGAAATG, product size 288 bp [5]). Quantification of the qRT-PCR fragments was carried out using the Eppendorf realplex^2^ Mastercycler eppgradient S (Eppendorf, Hamburg, Germany). Relative quantification was estimated by the ΔΔ*C*T method [20,21] with β-actin as a calibrator. The level of target gene expression was calculated by using 2^−ΔΔ*C*T^. Standard curves were prepared for the amplification specific efficiency correction and the efficiencies (E) were calculated according to the equation E = (10^−1^/m^−1^) × 100, where m is the slope of the linear regression model fitted over log-transformed data of the input cDNA concentrations versus CT values [20,21]. Efficiency represents the doubling each cycle, so 100% efficiency = 2.0, while 95% efficiency = 1.95.

In addition, PCR products were separated by agarose electrophoresis, visualized under ultraviolet illumination and product size was compared with a DNA ladder to confirm predicted size. Furthermore, RNA from several dilutions 100,000 cells (Luc^+^- and WT-mASCs in the dilutions of 1:1000; 1:200; 1:100; 1:50) were made to evaluate the sensitivity of the PCR assay for luciferase and the detection limit was documented with high-resolution gel electrophoresis.

### 4.5. In Vitro Imaging of Luc^+^-mASCs

Cells were seeded in triplicate in 96-well plates for the measurement of luciferase activity in cultured Luc^+^-mASCs. After 4 h, d-luciferin (VivoGlo^TM^ Luciferin, Promega, Mannheim, Germany) was added to each well at a final concentration of 0.5 mM. After 10 min, the luciferase activity was measured using an IVIS Lumina II Imaging system (Perkin Elmer, Rodgau, Germany). All data are expressed in photons/s (total flux) and analyzed with Living Image Software 4.0 (Caliper, Alameda, CA, USA).

### 4.6. In Vivo Bioluminescence Imaging

Mice were imaged at day 1, 3, and 6 post cell transplantation (day 2, 4, and 7 after induction of AKI, Figure S2) using an IVIS Lumina II Imaging system. The mice were anesthetized with Isoflurane and injected intraperitoneally with d-luciferin (VivoGlo^TM^ Luciferin, Promega, Mannheim, Germany) for in vivo imaging of the transplanted cells. Image acquisition was carried out from the dorsal and the ventral side, and started 10 min later by increasing acquisition time (1, 30, 60, 180, and 360 s). Regions of interest (ROIs) were drawn using Living Image software. Data were related to average background subtraction from control animals that received identical doses of luciferin to control the background photon emission in defined ROIs.

### 4.7. Statistical Analysis

The data were expressed as mean ± standard error of the mean (SEM). The comparison between groups was performed by one-way analysis of variance (ANOVA) and the Bonferroni post-hoc test using Prism 5 software (GraphPad, LaJolla, San Diego, CA, USA). *p* values < 0.05 were considered significant.

## Supplementary Materials

The following are available online at http://www.mdpi.com/1422-0067/19/9/2564/s1.

## Abbreviations

AKI	acute kidney injury
BLI	bioluminescence imaging
Cis	Cisplatin
i.*p*.	intraperitoneal
IV	intravenous
Luc^+^-mASCs	Luciferase transgenic adipose-derived stromal/stem cells
mASCs	adipose-derived stromal/stem cells
MSCs	mesenchymal stromal/stem cells
N-GAL	neutrophil gelatinase-associated Lipocalin
PCR	polymerase chain reaction
qRT	quantitative real-time reverse transcriptase
WT	wild type
