# Supplementary material for: Tracking of Adipose-Derived Mesenchymal Stromal/Stem Cells in a Model of Cisplatin-Induced Acute Kidney Injury: Comparison of Bioluminescence Imaging versus qRT-PCR
Source: Int J Mol Sci. 2018 Aug 29;19(9):2564. doi: 10.3390/ijms19092564 (PMC6165020; doi:10.3390/ijms19092564)
Supplement: Supplementary file 1 [file ijms-19-02564-s001.pdf]

Supplementary Materials:

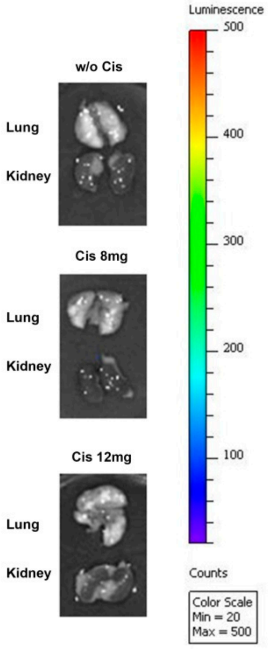

Fig. S1. Representative ex vivo Bioluminescence Imaging of lungs and kidneys on day 6 (endpoint). Cis = Cisplatin.

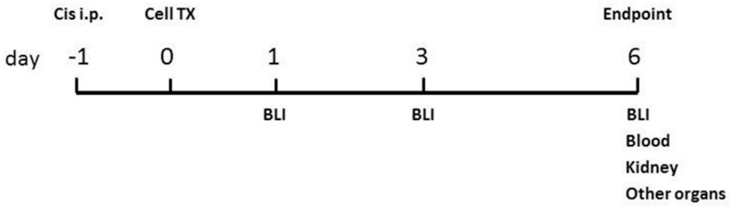

Fig. S2. Time table of the in vivo model. Cis = Cisplatin, TX= Transplantation, BLI = Bioluminescence Imaging.

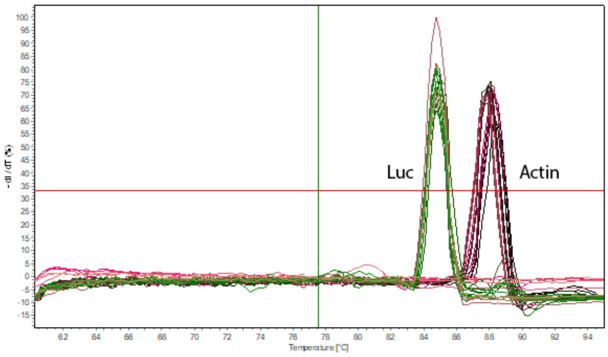

Fig. S2: Melting curves from Luc- and β-actin-qRT-PCR (using RNA from cultured Luc[+]-mASCs).
